# The relationship between the strength of religious faith and spirituality in relation to post-traumatic growth among nurses caring for COVID-19 patients in eastern Poland: a cross-sectional study

**DOI:** 10.3389/fpsyt.2023.1331033

**Published:** 2024-01-08

**Authors:** Grzegorz Józef Nowicki, Daria Schneider-Matyka, Iwona Godlewska, Andrzej Tytuła, Marzena Kotus, Monika Walec, Elżbieta Grochans, Barbara Ślusarska

**Affiliations:** ^1^Department of Family and Geriatric Nursing, Faculty of Health Sciences, Medical University of Lublin, Lublin, Poland; ^2^Department of Nursing, Faculty of Health Sciences, Pomeranian Medical University in Szczecin, Szczecin, Poland; ^3^Second Department of Anaesthesia and Intensive Therapy, Medical University of Lublin, Lublin, Poland; ^4^Head Chamber of Nurses and Midwives, Warszawa, Poland; ^5^Faculty of Human Sciences, University of Economics and Innovation, Lublin, Poland; ^6^Department of Anaesthesiological and Intensive Care Nursing, Medical University of Lublin, Lublin, Poland

**Keywords:** COVID-19, post-traumatic growth, religion, spirituality, nurses

## Abstract

**Introduction:**

The COVID-19 pandemic had forced intensive care unit (ICU) nurses to adapt to extreme conditions in a short period of time. This resulted in them experiencing extremely stressful situations. The aim of this study was to assess the relationship between post-traumatic growth (PTG) and religiosity and spirituality (R/S) among nurses caring for COVID-19 patients in intensive care during the pandemic.

**Materials and methods:**

120 nurses working in Lublin, eastern Poland, participated in the cross-sectional study. The questionnaire was made up of three standardised tools: The Post-Traumatic Growth Inventory, The Santa Clara Strength of Religious Faith Questionnaire, The Spiritual Attitude and Involvement List.

**Results:**

In terms of spirituality, the study group of nurses achieved the highest score in the Connectedness with Nature subscale (4.37 ± 1.07), while the strength of religious beliefs had a positive correlation with post-traumatic growth only in the Spiritual changes subscale (*r* = 0.422, *p* < 0.001). The following dimensions of spirituality were significantly correlated with post-traumatic growth in the multi-factor model that included religiosity and spirituality: Transcendent experiences, Spiritual activities, Meaningfulness, Acceptance, and Trust. We saw that increase in the assessment of the Transcendent experiences, Meaningfulness and Trust subscales significantly mirrors increase in post-traumatic growth, while increase in the assessment of the Spiritual activities and Acceptance subscales significantly mirrors decrease in post-traumatic growth. The above variables explained up to 44% of the dependent variable.

**Conclusion:**

Both religiosity and spirituality were significantly associated with post-traumatic growth in the group of ICU nurses, but spirituality appears to have played a larger role. Our findings support the value and significance of the development of spiritual and religious identity as a means of enhancing positive psychological changes in the face of traumatic events.

## 1 Introduction

Intensive care unit (ICU) nurses have one of the most stressful jobs involving specialised knowledge and extensive training in today’s workplace. During the COVID-19 pandemic, the workload of ICU nurses increased significantly, thus affecting their regular manner of providing care (1). In order to furnish patients with the necessary treatment and care, healthcare systems were also forced to undergo numerous reorganisations within their structures as a result of the rising demand for intensive care for COVID-19 patients. The phenomenon of an increase in the number of beds in the hospital wards hospitalising COVID-19 patients, as well as an improvement in the ability to diagnose and provide intensive care to COVID-19 patients was observed in Poland. In order to ensure the safety of health care workers and patients, managers of healthcare providers attempted to modify the work organisation, as well as the surroundings and hygienic conditions of the hospital wards intended for the diagnosis and treatment of patients with suspected or confirmed COVID-19 disease (2). The pandemic resulted in a severe decline in nursing care, which was associated with a lack of time, resources and necessary skills. Moreover, nurses experienced psychological and ethical stress due to the fear of not being able to provide essential nursing care (3). In comparison to doctors or other clinical staff, ICU nurses frequently reported experiencing higher levels of stress (4).

The COVID-19 pandemic forced ICU personnel to adapt to extreme conditions in a short period of time, resulting in experiencing outrageously stressful situations. The experience of ICU nurses caring for COVID-19 patients during the pandemic clearly demonstrates the psychological and physical effects of this challenging workplace (5). Even highly specialised and mentally strong nurses occasionally experienced psychological distress, such as post-traumatic stress disorder (PTSD) symptoms and increased levels of stress (6). Indeed, it must be underlined that ICU staff, especially nurses, experience higher levels of psychological and moral distress even in regular conditions, in contrast to non-intensive care units, as they frequently face more difficult tasks to perform, have to make difficult decisions, and must provide end-of-life care (7) – which is undoubtedly a feature of challenging conditions such as the pandemic (8).

There is a growing body of evidence that the traumatic life events experienced by health care workers (HCWs), particularly from the frontline of intensive care, that are related to trauma in the COVID-19 pandemic, may have many negative physical and psychological consequences or may serve as the foundation for post-traumatic growth (PTG) (9–11). One of the main goals of COVID-19 research is to identify the protective and risk factors for the psychological health of healthcare workers (12). However, the outcome of such experience, need not always be negative. PTG is defined as *positive psychological change experienced as a result of the struggle with highly challenging life circumstances* (13). Tedeschi et al. (14), for example, indicate that traumatic experiences can also be catalysts for positive change, which is consistent with the perception of improvement in characteristic personal resources, expressed in the conversation of resources theory (15).

Resilience is the human ability to adapt in difficult situations and ongoing major life stresses. Being resilient does not mean that people do not experience emotional upheaval, stress and suffering, but that they handle stress more positively. Such people are able to deal with their emotions and traumatic events as they are aware that difficult emotions and adversities do not last for very long (16). Religiosity and spirituality (R/S) can be a valuable resource for strengthening a person’s resilience. Spirituality and religiosity are defined as separate but overlapping constructs. Spirituality refers to an individual’s search for meaning and purpose in life (17). It is also defined as a dynamic and intrinsic aspect of being human, through which individuals seek ultimate meaning, purpose and transcendence, and experience relationships within themselves and with family, others, community, society, nature and all that is essential or sacred (18). Religiosity mainly refers to a set of rituals specific to a church institution and belief in God and other religious beliefs, while spirituality can be a non-denominational or denominational pursuit of personal development (19).

Having a sense of spirituality goes beyond having religiously awareness, it also helps in developing meaning in life and self-confidence in dealing with challenges (20). It should be noted that the personal spirituality of an individual nurse affects the spiritual nursing care they provide (21), since the spiritual wellbeing is linked to a number of favorable outcomes, such as a greater ability to tolerate the psychological and physical demands related to patients’ illness (22). Indeed, three major conclusions were drawn from a systematic review of 11 studies that demonstrated relationships between R/S and PTG: (1) Religiosity and spirituality are usually, although not always, beneficial to people in dealing with the aftermath of trauma; (2) traumatic experiences can lead to a deepening of religion or spirituality beliefs; (3) positive religious coping, religious openness, readiness to face existential questions, religious participation, and intrinsic religiousness are typically associated with post-traumatic growth (23). A systematic empirical review of the research on religion and COVID-19 conducted in the first year of the pandemic (24) supports the aforementioned conclusions. Other findings from the scoping review show that religiosity did not automatically aid ICU staff members in coping with moral distress or strengthen their resilience (25). Spirituality, on the other hand, described as a type of self-care (26), was mentioned as a resource for reducing moral distress (27, 28).

The COVID-19 pandemic outbreak led people to seek help in coping with the threat through religiosity and spirituality. According to Google search data, the word “prayer” was searched for more frequently than ever in 95 of the countries analysed. This trend was also observed in Poland (29). Poland’s society is among the most religious in Europe (30), with the Catholic Church constituting the country’s largest religious community (30). Throughout the last 20 years, more than 90% of all Poles have identified as believers, and nearly 50% of all Poles say they practice their religion regularly (at least once a week) (31). Annual statistics on church attendance indicate a marginally lower percentage of firm believers (approximately 40%), but this number is relatively stable and higher than in most European countries (32). Thus, religion continues to play a key role in the average Pole’s life, despite some indications of secularization within society (33).

In the Polish study by Boguszewski et al. (34), it was found that there was a rise in religious practice participation during the COVID-19 pandemic, as evidenced by the amount of time spent in prayer. According to the American study, 23% of HCWs thought that religiosity and spirituality was a way of dealing with COVID-19-related suffering (35). In the Italian study, Molteni et al. (36) found that those who had a family member diagnosed with COVID-19 were more religious following the diagnosis, attended religious services more frequently and prayed more often (via the Internet, radio, or television). However, despite the outbreak of the COVID-19 pandemic, a study conducted in the Netherlands to assess the frequency of prayer between 2017 and 2020 found no increase in religiosity as compared to the pre-pandemic period (37). The use of religiosity and spirituality to cope with the psychological suffering caused by the COVID-19 pandemic was therefore, highly impacted by a country’s cultural and social determinants (38).

The discrepancy of the current study results in the aforementioned area and the multitude of other factors that contribute to the PTG phenomenon among front-line nurses caring for COVID-19 patients (9) suggest that there is still a gap in the evidence, particularly in countries where a single religion is the predominant faith and the society considers itself to be deeply religious. Therefore, the aim of this study was to assess the relationship between post-traumatic growth and religiosity and spirituality among nurses caring for COVID-19 patients under intensive care during the pandemic.

## 2 Materials and methods

### 2.1 Study design and participants

This cross-sectional study was conducted between December 2022 and February 2023 using the paper and pen personal interview (PAPI) method and took part in Lubelskie province, Poland. In addition to a temporary hospital set up for the duration of the pandemic, the study involved the staff drawn from nine state hospitals, one of which is a children’s hospital. Each of these nine facilities set up a sub-department to treat COVID-19 patients during the pandemic. However, adults over the age of 18 who had the most severe course of the disease were admitted to the Independent Public Clinical Hospital No 1, while children with severe COVID-19 received specialised care at the University Children’s Hospital in Lublin. The aforementioned hospitals treated patients from the city of Lublin and the Lubelskie Province. Nurses working in the two Clinical Hospitals located in Lublin, eastern Poland during the time of the pandemic participated in the study. In order to obtain research material from nurses caring for the most severe COVID-19 patients, ICU staff were particularly taken into consideration. The study included nursing staff from the Department of Anaesthesiology and Intensive Care of the University Children’s Hospital in Lublin, as well as nursing staff from the 2nd Department of Anaesthesiology and Intensive Care and the Department of Infectious Diseases of the Independent Public Clinical Hospital No 1 in Lublin. The inclusion criteria included: (1) being employed as a nurse in the aforementioned hospital departments; (2) providing care for severe and critical COVID-19 patients, according to score 3 or 4 on the Modified Early Warning Score (MEWS) scale (39); (3) period of employment during the coronavirus pandemic, which is defined from 20th March 2020 until the end of the 5th wave of the COVID-19 pandemic; and (4) provision of informed consent for participation in the study. The exclusion criteria included: (1) the lack of employment during the COVID-19 pandemic, i.e., commencement of work after the end of the 5th wave of the pandemic, and (2) the lack of informed consent for participation in the study.

### 2.2 Data collection

A qualified nurse (IG and MW) distributed the questionnaires to the aforementioned departments throughout the study period. In December 2022, the questionnaires were left each week (every Monday). The completed forms were collected from a special box placed in the department by the designated research team member. In January and February 2023, the interviewer visited the departments every 2 weeks, including Mondays. The questionnaires were collected up until the point where the interviewer, who was responsible for reporting to the department where the study was conducted, twice failed to collect the completed questionnaires from the box. The respondents could ask questions during the interviewer’s visits. Eight nurses were on long-term sick leave out of the total 158 nurses who worked on given wards during the study period. There were 150 questionnaires distributed in total, 137 of which were completed. However, 17 questionnaires had to be rejected because of errors and missing answers, thus leaving 120 correctly completed questionnaires. According to the STROBE checklist, reporting of observational studies in epidemiology does not require sample size calculation (40).

### 2.3 Ethics approval

The study was conducted in accordance with the Declaration of Helsinki (updated in 2013). The research was approved by the Bioethics Committee of the Medical University of Lublin (Lublin, Poland) (KE-0254/73/2020), and all respondents gave their written informed consent for participation in the study.

### 2.4 Questionnaire

To achieve the study’s goal, a structured questionnaire comprised of three standardized tools and a researcher-made questionnaire was used. According to the questionnaire instructions, study participants were supposed to make an assessment based on the coronavirus pandemic-related events.

#### 2.4.1 Post-traumatic growth related to the coronavirus pandemic

The severity of post-traumatic growth in the study group of nurses was measured using the Post-Traumatic Growth Inventory (PTGI) by Tedesch and Calhoun (41) in the Polish adaptation of Ogińska-Bulik and Juczyński (42). The questionnaire in the Polish language version consists of 21 statements which describe different changes which occurred as the result of the experienced traumatic event. The changes are assessed on a 6-level scale from 0 – “I have not experienced this change,” to 5 – “I have experienced this change to a very great extent.” The tool in the Polish language version analyses the general indicator of the severity of PTG, as well as four factors affecting the development after experiencing a traumatic event: Changes in self-perception – as a result of an experienced trauma, a person notices new opportunities and perceives growth in personal strength; Changes in relating to others – greater sense of relation to others, increased empathy and altruism; Appreciation of life – changes in philosophy of life, change of priorities, greater appreciation of everyday life; Spiritual changes – better understanding of spiritual problems and an increase in religiosity. The overall score is the sum of all the factors referred to above. The higher the score, the higher the intensity of positive changes as a result of the experienced trauma. The Cronbach’s alpha coefficient in the current study for the general result was 0.97, for factors: Changes in self-perception – 0.93, Changes in relating to others – 0.95, Appreciation of life – 0.89 and Spiritual changes – 0.7.

#### 2.4.2 Assessment of the strength of religious faith and engagement

To assess the strength of religious faith and engagement, the Santa Clara Strength of Religious Faith Questionnaire (SCSRFQ) by Plante and Boccaccini (43) in the Polish adaptation of Wnuk (44) was used. The questionnaire is made up of 10 statements concerning religious beliefs. The respondent was asked to respond to a given statement using a four-point Likert scale ranging from 1. “I strongly disagree,” to 4. “I strongly agree.” The questionnaire assessment dimension is one-factor and measures the strength of religious beliefs regardless of the respondent’s religious denomination. The strength of religious faith is defined as faith in God, who is, thus, the source of consolation, inspiration, meaningfulness and purpose in life, and who serves as the central point for identification and shaping of a person’s sense of identity. The Cronbach’s alpha coefficient in the current study amounted to 0.97.

#### 2.4.3 Assessment of spirituality

In order to measure spirituality, the Spiritual Attitude and Involvement List (SAIL) by de Jager Meezenbroek et al. (45) in the Polish adaptation by Deluga et al. (21) was used. The questionnaire in the Polish language version is made up of 26 statements. The respondent was asked to indicate to what extent the thesis contained therein applies to him or her. In the statements from 1 to 18, the respondent could choose from 1 – “Not at all,” to 6 – “To a very great extent,” while in statements from 19 to 26, the respondent was asked to indicate: 1 – “Never,” to 6 – “Very often.” The explanatory factor analysis, based upon the SAIL Polish adaptation, identified a six-factor structure. The subscales of the questionnaire included: Transcendent experiences (going beyond reality, reaching another level of human experience and senses, experiencing the Absolute Power/God/Higher Force), Spiritual activities (effort and engagement in the world of values, communication with the Absolute Power/God/Higher Force), Connectedness with Nature (connection with the natural world, admiration for the universe), Meaningfulness (sense of meaning and value of life, seeing the value of life in sacrificing one’s own life for the sake of others), Acceptance (ability to deal with a variety of situations, acceptance of harsh realities of life) and Trust (trust in life, feeling of powerlessness over all aspects of life, faith in God’s providence). The Cronbach’s alpha coefficient in the current study was: Transcendent experiences – 0.83, Spiritual activities – 0.7, Connectedness with Nature – 0.78, Meaningfulness – 0.86, Acceptance – 0.7 and Trust – 0.71.

#### 2.4.4 Sociodemographic variables

The respondents were asked to provide sociodemographic data in the subsequent questions. The questions concerned the following variables: age, gender, place of residence, marital status, pre-graduate education, post-graduate education and years of service as a nurse.

### 2.5 Statistical analyses

Continuous variables are expressed as mean (M) and standard deviation (SD) or median (interquartile range, IQR) as appropriate. The Shapiro–Wilk test was applied to assess conformity with a normal distribution. Relationships between religious faith, spiritual attitudes and involvement and post-traumatic growth were examined by Pearson correlation and multivariable linear regression. *p* values <0.05 was considered statistically significant. All statistical analyses were performed using IBM software (released in 2019) and IBM SPSS Statistics for Windows, Version 26.0. (IBM, Armonk, NY, United States).

## 3 Results

### 3.1 Characteristics of participants

Table 1 presents the characteristics of the study group. A total of 120 nurses took part in the study. The mean age in the study group was 43.1 ± 10.6 years. The majority of respondents (82.5%, *n* = 99) consisted of women who lived in an urban area, and were in a relationship (79.17%, *n* = 95). The median length of service of the nurses was 20.5 years (Q1 = 8.5; Q3 = 30).

**Table 1:** Sociodemographic analysis of the study group.

Characteristic	Group	Study group
Age [year]^b^		43.1 ± 10.6
Gender^a^	Woman	99 (82.5)
	Man	21 (17.5)
Place of residence^a^	City	89 (74.17)
	Village	31 (25.83)
Marital status^a^	In a relationship	95 (79.17)
	Single	25 (20.83)
Education^a^	Registered nurse (secondary nursing school – lyceum)^1^	15 (12.5)
	Registered nurse with bachelor’s degree in nursing	40 (33.33)
	Registered nurse with master’s degree in nursing	65 (54.17)
Postgraduate education^c^	Postgraduate diploma	1 (0.83)
	Specialist training course	81 (67.5)
	Qualification course	30 (25)
	Specialist course	8 (6.67)
Years of experience [years]^a^		20.5 (8.5–30)

Data presented as: ^a^ *n* (%), ^b^ mean ± SD, or ^c^ median (Q1–Q3); ^1^ Secondary Nursing School (Lyceum) – Nursing education system in Poland before 1999.

### 3.2 Distribution of the analysed features according to scales PTGI, SCSORF, and SAIL

Table 2 shows the results of the respondents as mean scores on the scales used in the study. The RTG total score in the study group amounted to 2.74 ± 1.21. The highest mean score in the study group concerned the Appreciation of life factor (3.14 ± 1.4). The second highest rated factor was Changes in relating to others (2.74 ± 1.33) and the third was Changes in self-perception (2.72 ± 1.21).

**Table 2:** Distribution of the analysed features in scales.

Scales	M ± SD
PTGI – Total score	2.74 ± 1.21
PTGI – Changes in self-perception	2.72 ± 1.21
PTGI – Changes in relating to others	2.74 ± 1.33
PTGI – Appreciation of life	3.14 ± 1.4
PTGI – Spiritual changes	2.25 ± 1.43
SCSORF – Strength of religious faith and engagement	24.79 ± 8.55
SAIL – Transcendent experiences	3.44 ± 1.06
SAIL – Spiritual activities	3.64 ± 0.93
SAIL – Connectedness with Nature	4.37 ± 1.07
SAIL – Meaningfulness	4.23 ± 0.96
SAIL – Acceptance	4.1 ± 0.8
SAIL – Trust	3.85 ± 0.97

M, mean; SD, standard deviation; PTGI, The post-traumatic growth inventory; SCORF, The Santa Clara strength of religious faith questionnaire; SAIL, the spiritual attitude and involvement list.

The mean score for strength of religious faith and engagement in the study group of nurses was 24.79 ± 8.55. Taking into account spirituality, the study group of nurses achieved the highest score on the Connectedness with Nature subscale (4.37 ± 1.07) and lowest on the Transcendent experiences subscale (3.44 ± 1.06).

### 3.3 The relationship between post-traumatic growth and strength of religious belief and spirituality

Table 3 reveals the relationship between the PTGI subscales and religiosity and spirituality. A significant positive correlation between strength of religious belief and the PTGI Spiritual Change subscale was observed. There was no significant relationship with the other PTGI subscales. In terms of the relationship between spirituality and PTG, the following SAIL subscales were found to have a significant and positive correlation: Transcendental Experiences, Meaningfulness, Acceptance and Trust with an overall PTG score and all PTGI subscales. Additionally, both the SAIL Spiritual Activity and Connectedness with Nature subscales were significantly and positively correlated with the PTGI Spiritual Changes subscale (*r* = 0.437, *p* < 0.001 vs. *r* = 0.237, *p* = 0.009), and the latter was also positively connected with the Appreciation of Life subscale (*r* = 0.236, *p* = 0.009).

**Table 3:** The relationship between the PTGI subscales and the strength of religious belief and spirituality.

Variable		PTGI – Total score	PTGI – Changes in self-perception	PTGI – Changes in relating to others	PTGI – Appreciation of life	PTGI – Spiritual changes
SCSORF – Strength of religious faith and engagement	*r*	0.136	0.023	0.161	0.118	0.422
	*p*	0.139	0.799	0.079	0.201	<0.001
SAIL – Transcendent experiences	*r*	0.511	0.499	0.453	0.435	0.535
	*p*	<0.001	<0.001	<0.001	<0.001	<0.001
SAIL – Spiritual activities	*r*	0.177	0.100	0.172	0.137	0.437
	*p*	0.053	0.277	0.060	0.136	<0.001
SAIL – Connectedness with Nature	*r*	0.188	0.144	0.168	0.236	0.237
	*p*	0.039	0.117	0.067	0.009	0.009
SAIL – Meaningfulness	*r*	0.491	0.453	0.473	0.518	0.346
	*p*	<0.001	<0.001	<0.001	<0.001	<0.001
SAIL – Acceptance	*r*	0.275	0.249	0.242	0.307	0.265
	*p*	0.002	0.006	0.008	0.001	0.003
SAIL – Trust	*r*	0.325	0.273	0.331	0.324	0.299
	*p*	<0.001	0.003	<0.001	<0.001	0.001

*r*, correlation coefficient; PTGI, the post-traumatic growth inventory; SCORF, The Santa Clara strength of religious faith questionnaire; SAIL, the spiritual attitude and involvement list.

### 3.4 The relationship between post-traumatic growth and strength of religious belief and spirituality – a multivariate analysis

Table 4 shows the relationship between the overall assessment of post-traumatic growth and the strength of religious faith and spirituality. The model was statistically significant (*F* = 12.734, *p* < 0.001). The following spiritual dimensions were found to be significantly correlated with the overall PTG score: Transcendent experiences, Spiritual activities, Meaningfulness, Acceptance and Trust. Here, increase in the assessment of the Transcendent experiences, Meaningfulness and Trust subscales mirrors increase PTG. However, increase in the assessment of the Spiritual activities and Acceptance subscales mirrors decrease in PTG. The model’s variables explained 44% of the variation in PTG (*R*^2^ = 0.443).

**Table 4:** The relationship between post-traumatic growth and strength of religious belief and spirituality.

Variables	*b*	95% CI	*p*
SCORF – Strength of religious faith and engagement	0.040	(−0.571; 0.651)	0.896
SAIL – Transcendent experiences	13.102	(8.225; 17.979)	<0.001
SAIL – Spiritual activities	−7.149	(−14.021; −0.276)	0.042
SAIL – Connectedness with nature	−2.547	(−7.447; 2.354)	0.305
SAIL – Meaningfulness	14.920	(8.821; 21.019)	<0.001
SAIL – Acceptance	−14.445	(−23.468; −5.422)	0.002
SAIL – Trust	6.393	(0.866; 11.920)	0.024

*b*, linear regression coefficient; SCORF, The Santa Clara strength of religious faith questionnaire; SAIL, the spiritual attitude and involvement list.

## 4 Discussion

Research on identifying factors related to the PTG experience among various disciplines due to the COVID-19 pandemic is developing quickly. Wu et al. (46) conducted a systematic review and meta-analysis of 26 studies in the general population and discovered that between 10 and 77.3% of participants experienced PTG. The wide range of trauma experienced by participants in the studies likely contributed to the variations in PTG experiences. As supported by studies conducted, among others, in Korea (47), China (10), Hong Kong (48), and Australia (49), the PTG experience of nurses caring for patients with COVID-19 is largely comparable to that of our study, that is, moderate. In contrast, lower results were observed in other studies such as among Chinese and Taiwanese nurses (50) or in a study conducted among HCWs in Spain (51). Finding PTG factors is especially crucial for HCWs. In this study, we assessed the relationship between R/S and PTG in a group of ICU nurses caring for the most severe COVID-19 patients. Our findings demonstrated that R/S was positively correlated with PTG, but spirituality had a greater impact on PTG in the study group of nurses. Our study results showed how important R/S is for dealing with traumatic events like the COVID-19 pandemic.

A systematic analysis of 27 studies on the factors linked to PTG in HCWs found several demographic, individual, interpersonal and environmental factors (52). The benefits of the PTG experience include making sense of loss (53), the development of wisdom (54), and enhanced purpose and meaningfulness of traumatic experience that can last for up to 10 years after the trauma (55). Cui et al. (10) discovered that PTG levels were higher in front line nurses who were highly self-confident, highly risk-aware, and deliberate in their decision-making. In turn, Zhang et al. (56) found that self-efficacy can positively predict the PTG level among nurses. Chang et al. (57) discovered that positive self-compassion, wisdom, age, and deliberate rumination were the most important predictors of PTG development in nurses in intensive care units.

Wilson and Boden (58) noted that among the factors that contribute to PTG, religious and spiritual beliefs may play a prominent role in reaction to traumatic events. R/S has a wide range of potential advantages, including the ability to overcome traumatic events by giving one a sense of meaning and purpose in life, harmony and inner peace, and the conviction that they are being cared for by a Higher Power (59). Disasters, pandemics, and other traumatic events cast doubt on our world-views, and those who identify as religious or spiritual often turn to their faith to make sense of their suffering (60). According to research, it is not the devoutness of survivors that determines their post-disaster recovery, but rather how they engage in their faith (61). The results of our research seemed to confirm the above statement. The following SAIL subscales were found to have a positive correlation with spirituality, as was the overall PTG score: Transcendent experiences, Meaningfulness, Acceptance and Trust. The SAIL Spiritual Activity subscale was positively correlated with PTGI Spiritual Changes subscale. In the Spiritual Changes subscale, it was found that religious faith only had a positive correlation with PTGI. Although the strength of religious belief was not significantly related to PTG in the multivariable model in our study, some SAIL subscales explained 44% of the variability in PTG. According to the study results, from a relational perspective, R/S engagement can be understood dialectically, including movements toward spiritual dwelling or spiritual seeking. Spiritual dwelling includes practices that foster security, communal affiliation, affect regulation, and spiritual grounding. Spiritual seeking involves grappling with uncertainties and showing a willingness to question and reshape personal views and an appreciation for paradox and complexity (62). Therefore, spirituality, when facing post-traumatic stress, may have a significant defensive function by improving the accessibility to one’s personal psychological resources. It can also link survivors with social capital to cope with it and create valuable spiritual support networks (60, 63).

Our study results regarding the impact of religiosity and spirituality on PTG related to nursing care for COVID-19 patients should be discussed in light of similar studies conducted in non-HCW study samples. Like our study, other authors have also reported that variables related to religiosity and spirituality can assist individuals in managing the COVID-19 effects. Thus, religious and spiritual identity can provide meaning and resilience in both healthcare practice and adversity management (64, 65). Research by Willey et al. (66), Henson et al. (67), and Shigemoto (68) demonstrated, for example, that higher levels of spirituality and religiosity mediate the beneficial effects of ethnicity on PTG growth and predict higher levels of post-traumatic growth. Zhang et al. (69) in a cross-sectional study sample of adults recruited through an online platform, found that spiritual fortitude (SF), understood as one’s ability to consistently draw on spiritual and religious resources to cope with negative emotions in the face of stressors, buffered the relationship between loss of resources and the occurrence of the COVID-19 pandemic-related symptoms, such as depression, anxiety and PTSD. The authors concluded that the relationship between the loss of resources and mental health-related symptoms was weaker for those with high SF levels than for those with low SF levels. Prieto-Ursúa et al. (70), in the study conducted among Madrid residents, found that greater identification of R/S was linked to increased post-traumatic growth.

Other researchers analysed the effect of religion on perceptions of positive changes related to the COVID-19 pandemic. Lucchetti et al. (71) conducted a cross-sectional study with 485 participants from across Brazil in May 2020. The authors concluded that there was a high use of religious and spiritual beliefs during the COVID-10 pandemic and this use was associated with better mental health outcomes. They observed that lower levels of worrying were associated with greater private religious activities, religious attendance, spiritual growth and with an increase in religious activities. Lower levels of fear were associated with greater private religious activities and spiritual growth and, lower levels of sadness were associated with spiritual growth. Other researchers did not find a connection between religiosity and the COVID-19-related growth or positive changes during the pandemic. Chen et al. (72) compared the study results in a Chinese adult community sample before and during the COVID-19 pandemic, and concluded that meaning in life scores attributed to religious belief during the pandemic were lower than in a 2017 sample. However, Kye et al. (73), in a study conducted among South Korean adults, found that public trust in religious organisations sharply decreased during the COVID-19 pandemic, which may also be related to less frequent participation in religious practices. Other studies found a more complex relationship between religion and change perceptions. Counted et al. (74) conducted a study on a sample of Colombians and South Africans. According to the study results, the Colombian respondents characterised by a low level of hope had higher levels of well-being when positive religious coping was higher, while in the case of the South African respondents, their well-being was higher among participants who reported lower levels of hope and when negative religious coping was lower.

Although our findings clearly show that various dimensions of religiosity and spirituality are positively correlated with PTG, the precise mechanisms and other determinants of their development require further investigation. This should also be considered in light of Poland’s broader sociocultural aspects, specifically the decline in religious practice during and after the pandemic. However, when compared to other European countries, this rate remains relatively high (34). During the COVID-19 pandemic, traumatic events at nurses’ workplaces involving risking their own health and life, contact with patients’ suffering, or death resulted in an increase in spirituality assessment in the Transcendent experiences, Meaningfulness and Trust subscales, which led to an increase in the level of post-traumatic growth. However, other spirituality subscales, i.e.: Spiritual activities and Acceptance decreased the level of post-traumatic growth. Our study results indicate a two-pronged impact on PTG nurses’ experience, but it is unclear how effective they will be in generating the potential for this growth. Finally, our study demonstrates how to look for moderators and mediators of these correlations.

### 4.1 The strengths and limitations

The advantages of this study need to be taken into account. Firstly, our study was conducted with a group of nurses caring for the most severe COVID-19 patients, thus who were most vulnerable to psychological consequences. Secondly, we assessed the strength of religious faith and spirituality using validated and recognized scales. Thirdly, by employing separate tools to assess the strength of religious faith and spirituality, we were able to maintain the conceptual diversity of these two constructs, explaining their separate involvement in PTG. Fourthly, our research was carried out at a time when COVID-19 was no longer causing as much concern as it did when it started, and the staff had the tools and knowledge to counter the virus. We are currently in the period of mental recovery following the COVID-19 pandemic.

However, our research has some limitations. Firstly, our study is cross-sectional and does not demonstrate cause-and-effect or time-and-effect relationships, and as literature has shown, the psychological and spiritual effects of trauma may change in the face of different natural disasters (75, 76). Our findings, however, were primarily descriptive and were aimed at a professional group of nurses caring for COVID-19 patients. Secondly, the results from the small sample size may make it difficult to interpret the findings. Thirdly, the data was provided by ICU nurses caring for the most severe COVID-19 patients. Such individuals were more likely to have experienced greater trauma than nurses caring for patients experiencing a milder course of the COVID-19 disease. Fourthly, our study group is mostly made up of women, which reflects the fact that nursing is primarily a female-dominated profession. Nevertheless, women are more religious than men, which may have affected the R/S scoring in our study. Fourthly, because Polish society has historically been deeply religious and predominately Roman Catholic, research in a more secular and multi-religious society is thus required. Despite all these limitations, this study shows that among nurses who worked during the pandemic, religiosity and spirituality played a predictive role as factors correlated with PTG.

## 5 Conclusion

In summarizing the study’s preliminary findings, it should be noted that while spirituality and engagement in the Transcendent Experiences, Meaningfulness, Acceptance, and Trust subscale significantly positively correlated with the overall PTG score, as well as individual subscales, the strength of religious beliefs positively correlated with PTG only in the Spiritual Changes subscale. Additionally, spirituality in the Spiritual Activity and Connectedness with Nature subscales was significantly and positively correlated with the Spiritual Changes subscale, and the Connectedness to Nature subscale was also positively correlated with the Appreciation of Life subscale. In a multivariate analysis, the variables in the model explained 44% of the variability in PTG: increase in the assessment of the Transcendent experiences, Meaningfulness and Trust subscales, mirrors significant increase in PTG. However, increase in the assessment of the Spiritual activities and Acceptance subscales mirrors decrease in PTG.

Our study supports the usefulness and significance of a spiritual and religious identity development approach as a means of promoting positive psychological change that may arise from the highly difficult working conditions that nurses encounter that can manifest as trauma. Valuing healthcare personnel’s cultural and religious identity and encouraging self-reflective activities, such as mindfulness and meditation, as well as religious practice can help to promote post-traumatic growth. Professional organisations should also provide structured and supportive opportunities for staff members to reflect on the effects of trauma, encouraging them to think about how their spiritual, religious, and cultural identities are a source of strength and community for their personal and professional development. This prompts consideration on the implementation of spiritual support as a way to mitigate the effects of the pandemic or any encounter with acute trauma.

## Data availability statement

The raw data supporting the conclusions of this article will be made available by the authors, without undue reservation.

## Ethics statement

This study was approved by the Bioethics Committee of the Medical University of Lublin (decision number: KE-0254/73/2020). The studies were conducted in accordance with the local legislation and institutional requirements. The participants provided their written informed consent to participate in this study.

## Author contributions

GN: Conceptualization, Data curation, Formal analysis, Funding acquisition, Investigation, Methodology, Resources, Supervision, Validation, Visualization, Writing – original draft, Writing – review & editing. DS-M: Funding acquisition, Validation, Writing – review & editing. IG: Conceptualization, Investigation, Project administration, Writing – review & editing. AT: Data curation, Methodology, Project administration, Writing – review & editing. MK: Formal analysis, Writing – original draft. MW: Project administration, Validation, Writing – review & editing. EG: Funding acquisition, Resources, Supervision, Writing – review & editing. BŚ: Funding acquisition, Resources, Supervision, Writing – review & editing.
